# Ancestral reconstruction of reproductive traits shows no tendency toward terrestriality in leptodactyline frogs

**DOI:** 10.1186/s12862-015-0365-6

**Published:** 2015-05-20

**Authors:** Elisa Barreto Pereira, Rosane Garcia Collevatti, Marcelo Nogueira de Carvalho Kokubum, Núbia Esther de Oliveira Miranda, Natan Medeiros Maciel

**Affiliations:** Laboratório de Genética & Biodiversidade, Instituto de Ciências Biológicas, Universidade Federal de Goiás (UFG), Campus Samambaia, 74001-970 Goiânia, Goiás Brazil; Laboratório de Herpetologia e Comportamento Animal Departamento de Ecologia, Instituto de Ciências Biológicas, Universidade Federal de Goiás, Campus Samambaia, 74001-970 Goiânia, Goiás Brazil; Unidade Acadêmica de Ciências Biológicas/CSTR, Universidade Federal de Campina Grande (UFCG), 58704-300 Patos, Paraíba Brazil

**Keywords:** *Adenomera*, *Leptodactylus*, Reproductive mode, Stochastic inference, Trait correlation

## Abstract

**Background:**

Traditionally, the evolution of terrestrial reproduction in anurans from ancestors that bred in water has been accepted in the literature. Still, the existence of intermediate stages of water dependency, such as species that lay eggs close to water (e.g., in burrows) instead of in bodies of water, supports the hypothesis of an ordered and gradual evolution in the direction of a more terrestrial form of reproduction. However, this conventional view has recently been challenged for some anurans groups. Leptodactylinae frogs are a remarkable example of anurans with an outstanding diversity in terms of reproductive features, with distinct water dependency among lineages. Here, we tested the hypothesis of a gradual and ordered tendency towards terrestriality in Leptodactylinae, including the existence of obligatory intermediate stages, such as semi-terrestrial reproductive strategies. We also addressed the association between reproductive modes and the morphological and ecological features.

**Results:**

An ancestral reconstruction analysis indicated that even though shifts from aquatic to terrestrial breeding occurred throughout the history of *Leptodactylus* and *Adenomera*, shifts from terrestrial to aquatic reproduction happened at almost the same frequency. Our results also demonstrated that reproductive modes for semi-terrestrial tadpoles were not necessarily an intermediate form between aquatic and terrestrial breeds. Correlations among reproductive modes and other life-history traits suggested that tadpole environment, clutch size, nuptial spines, and egg pigmentation were co-evolving and driven by water dependency.

**Conclusions:**

Our results found no evidence of evolutionary tendencies toward terrestriality in Leptodactylinae. We found reversals from terrestrial to aquatic tadpole development and no evidence of obligatory intermediate stages, such as semi-terrestrial reproductive strategies. We also found correlations between reproductive modes and other life-history traits driven by water dependence. Aquatic reproductive modes are associated with higher clutch sizes, lentic waters, and the presence of nuptial spines and egg pigmentation.

**Electronic supplementary material:**

The online version of this article (doi:10.1186/s12862-015-0365-6) contains supplementary material, which is available to authorized users.

## Background

The evolution of life-history traits in different lineages is a major question in the field of evolutionary biology [1], especially because traits may drive the speciation process [2,3]. Indeed, niche-related traits, for example, could diverge, producing reproductive isolation between two populations. As a result, this leads to ecological speciation. Coevolution is another interesting topic that determines the process of evolutionary change in a trait triggered by other trait in a lineage. Trait evolution among lineages could occur through reciprocal evolution between interacting species, driven by natural selection [4].

Analyses of character evolutionary history, such as ancestral state reconstruction (reviewed by [5]) and stochastic character mapping [6], are powerful methods for studying the origin and maintenance of phenotypic diversity [2,3]. Moreover, the analysis of character associations may provide important clues regarding the coevolution between life-history traits [7]. The Bayesian approach to investigating patterns of ancestral states is considered a more powerful method compared to other methods, such as parsimony. Parsimony is unable to consider more than a single change along the branch on a cladogram and cannot couple with evolutionary time and amount of character change [8].

In amphibians, the evolution of reproductive features is still not completely understood due to the remarkable diversity of lifestyles, from purely aquatic to arboreal and fossorial [9]. Anuran reproductive features are classified by reproductive modes based on oviposition, development, stage and size of hatchling, and parental care [9,10]. Each reproductive mode classification in frogs is assigned a number according to the dependence on water for reproduction. For instance, mode number 1 is associated with frogs that deposit their eggs in water where exotrophic tadpoles develop. Frogs presenting reproductive mode number 17 lay eggs in excavated nests where tadpoles live in early stages and subsequently complete their development in ponds or streams. Direct development frogs present reproductive mode number 23 (see details in [9]).

Shifts from aquatic to terrestrial breeding have occurred repeatedly and independently in many vertebrates [11]. Traditional knowledge claims that the evolution of terrestrial reproduction in anurans occurred from ancestors that bred in water [10,12], especially because the aquatic mode is the most representative of exotrophic tadpoles [9] and was probably the ancestral state for anurans [13]. In addition, the existence of intermediate stages of water dependency, such as species that lay eggs close to water (e.g., in burrows) instead of inside bodies of water, supports the hypothesis of an ordered and gradual evolution in the direction of a more terrestrial reproduction [12,14]. Nonetheless, Gomez-Mestre et al. [13] has recently challenged this conventional view by demonstrating the lack of intermediate stages in some groups, as well as the evolution of direct development from both terrestrial and aquatic reproductive modes.

In addition, changes between aquatic and terrestrial breeding may occur in conjunction with modifications of morphological and other ecological features [15,16], providing opportunities for coevolution between traits. Even though reproductive modes are frequently studied, the only well-known associations that are commonly tested show: (i) negative correlations between ovum and clutch size (number of eggs per spawning); (ii) positive correlations between ovum size and hatchling dimensions; and (iii) positive correlations between clutch volume, egg size and female body size within a given reproductive mode (see [12]). Under a cladistic perspective, a recent study shed light on some unexplored associations, such as the correlation of terrestrial reproduction with reduced clutch and adult size, and with parental care [13]. However, little is known about other traits that may be correlated with reproductive modes, such as tadpole and adult morphological characteristics, with the exception of body size.

Amphibian systematics has undergone pronounced changes over the past decade (e.g., [17,18]). The genus *Leptodactylus,* the most diverse of the Leptodactylidae, contains 75 species distributed throughout North America (southern Texas), as well as Central and South America. Formerly, the genus was assembled in five groups based on behavioral, morphological and ecological features [15]: the *Leptodactylus ocellatus* group, now referred to as the *L. latrans* group [19], the *L. melanonotus* group, the *L. pentadactylus* group, the *L. fuscus* group and the *L. marmoratus* group. However, since Heyer’s [15] suggestion that the group *Leptodactylus marmoratus* was not closely related to the other groups, the phylogenetic position of the group has been discussed, leading to its placement in a different genus, *Adenomera* [20]. Recent molecular data confirm *Adenomera* as a natural group with a single common ancestor [18,21]. Distributed throughout almost all of South America, the genus is currently comprised of 18 species. This number is known to be underestimated, however, due to the occurrence of cryptic species [22,23].

**Figure 1:**
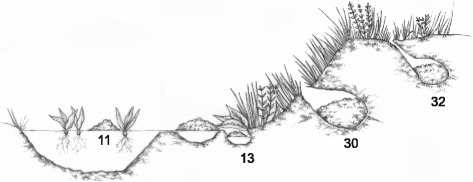
Schematic drawings representing known reproductive modes in Leptodactylinae. Mode 11 includes species that produce floating foam nests in ponds with exotrophic tadpoles; Mode 13 also presents exotrophic tadpoles, but with foam nests placed in water accumulated in constructed basins; Mode 30 groups species that have foam nests that are placed inside a subterranean chamber and after a period of development, the tadpoles float to the bodies of water; Mode 32 is the most terrestrial one, with endotrophic tadpoles (developing entirely in subterranean chambers using only the yolk as a source of energy). Illustrated by Vinícius Yano.

*Leptodactylus* and *Adenomera* (Anura: Leptodactylidae: Leptodactylinae) are good models for understanding the patterns and processes of the evolutionary history of reproductive traits. These foam-nesting species present at least four different reproductive modes, varying in oviposition and biology of the larvae. The diversity of reproductive modes for *Leptodactylus* and *Adenomera,* as well as its association with species in the phenetic groups of *Leptodactylus* and in the *Adenomera,* has led to the prediction of a gradual evolutionary tendency of evolutionary lineages, from a more aquatic to a more terrestrial breeding. This also suggests the presence of obligatory intermediate stages of water dependence to reproduction. Heyer [15] hypothesized that the *Leptodactylus melanonotus* and *L. latrans* groups had the most primitive reproductive modes, with higher water reliance (Figure 1, mode number 11). The *Leptodactylus pentadactylus* group would represent the first step towards terrestriality, with eggs placed in the water accumulated in basins constructed by males (Figure 1, mode number 13), followed by the *L. fuscus* group, in which eggs are placed inside subterranean chambers that are also constructed by males (Figure 1, mode number 30). Finally, *Adenomera* (formerly the *L. marmoratus* group) would represent the most derived reproductive mode, with a lower dependency on water for reproduction, since some species are known to have endotrophic tadpole development in subterranean chambers constructed by males (Figure 1, mode number 32). However, Heyer [15] suggested that *Adenomera* was an independent lineage, and postulated that the evolutionary shift to terrestrial reproduction in leptodactylines occurred twice, once in the *Leptodactylus* ancestor and another in the *Adenomera* ancestor. Although this hypothesis of a gradual increase of terrestriality in some Leptodactylinae frogs has never being tested, it has repeatedly been cited in the literature (e.g., [9,24-28]). Recently, some authors have raised questions concerning this hypothesis [29,30].

Here, we studied the evolution of life-history traits among Leptodactylinae lineages by reconstructing ancestral states, mapping character changes and testing the correlation among six characteristics. In this study, we tested: (1) the hypothesis of the tendency towards terrestriality, with shifts from aquatic to terrestrial breeding and the existence of obligatory intermediate stages; and (2) the association between reproductive modes and morphological and ecological features that are potentially related to water dependency.

## Results

### Phylogeny estimation

The combined dataset alignment consisted of a fragment of 1,526 base pairs (Table 1). The third codon position of *cytB* was excluded from the final alignment due to high saturation (Additional file 1). Some hypervariable regions with several indels were excluded from the 12S and 16S sequences because of the ambiguous alignments they generated. For both the 12S and 16S datasets, the best evolutionary model was GTR+I+G, whereas for the *cytB* fragment, it was TIM2+I+G. Finally, for the *Rhod* fragment, the best evolutionary model was TPM3uf+I+G (Table 1).

**Table 1:** **Sequence characterization and evolutionary model used in phylogenetic analyses for 35 Leptodactylinae species**

	**16S**	**12S**	**Cytochrome B**	**Rhodopsin 1**
**Original length (bp)**	517	435	405	330
**Final length (bp)**	503	423	270	330
**Base frequencies**				
**%A**	0.314	0.308	0.211	0.234
**%C**	0.230	0.262	0.216	0.283
**%G**	0.210	0.203	0.230	0.195
**%T**	0.246	0.227	0.343	0.288
**Parsimony informative characters (PIC)**	116	107	28	44
**PIC without outgroup**	108	101	27	27
**Best fit model**	GTR+I+G	GTR+I+G	TIM2+I+G	TPM3uf+I+G
**Model likelihood**	2,904.84	2,865.12	868.58	1,081.27

**Figure 2:**
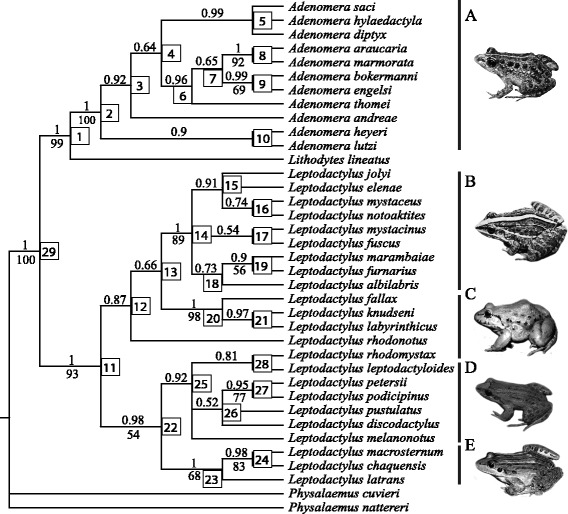
Phylogenetic relationships among Leptodactylinae, based on the 50% majority rule consensus cladogram reconstructed using the Bayesian analysis. Numbers inside squares represent clade numbers. Numbers above nodes are clade *posteriori* probability, and below nodes are bootstrap supports for the maximum parsimony analysis. A: *Adenomera saci* (*Adenomera* genus); B: *Leptodactylus fuscus* (*L. fuscus* group); C: *L. labyrinthicus* (*L. pentadactylus* group); D: *L. podicipinus* (*L. melanonotus* group); and E: *L. latrans* (*L. latrans* group). Photos: A, Pedro Peloso, B and D, Ariovaldo Giaretta, and C and E, Antonio Sebben.

The Bayesian analysis resulted in a monophyletic and highly supported *Adenomera* clade with *Lithodytes lineatus* as a sister species (Figure 2). *Adenomera heyeri* and *A. lutzi* comprises a sister clade of all other *Adenomera* species sampled. The *Leptodactylus* species also formed a highly supported monophyletic group, subdivided into two major clades (Figure 2).

The maximum parsimony analyses produced 50 of the most parsimonious trees with 1,435 steps. The strict consensus tree had 1,472 steps (CI = 0.41, RI = 0.56) and also showed *Lithodytes lineatus* as a sister species to the monophyletic genus *Adenomera*. The parsimony analyses generated a consensus tree similar to the Bayesian analysis, but also presented some polytomies.

### Ancestral state reconstruction, character mapping, and correlation

**Figure 3:**
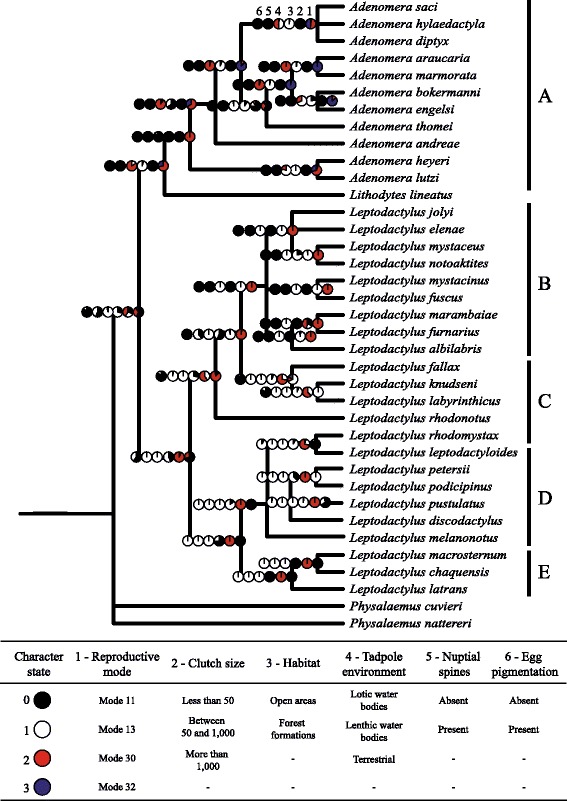
Ancestral state representation of six life-history traits reconstructed for 35 Leptodactylinae species using stochastic inferences. Pie charts indicate the probability of each character state. Clade numbers are indicated at the nodes (inside squares) of the Bayesian cladogram (Figure 2). Only the ancestral state probability of the clades in the 50% majority-rule consensus cladogram reconstructed using Bayesian analysis are indicated here. See Additional file 2 for the probability of each character state in each possible clade, as pointed out by the Bayesian analysis. A: *Adenomera* genus; B: *Leptodactylus fuscus* group; C: *L. pentadactylus* group; D: *L. melanonotus* group; and E: *L. latrans* group.

The Bayesian character state reconstruction indicated that reproductive mode 11, in which eggs are placed in floating foam nests directly on the top of water, was the most probable ancestral state of the most recent common ancestor of Leptodactylinae (node 29, Figure 3). This reproductive mode was also the inferred ancestral state of *Lithodytes* + *Adenomera* (node 1) and of *Leptodactylus* (node 11). While reproductive mode 11 had one origin, modes 13 and 32 originated twice and mode 30 originated at least three times (Figure 3). Reproductive mode 13 is characterized by the deposition of foam nests on water that has accumulated in a constructed basin. In mode 30, parents produce foam nests where eggs and early larval stages develop in subterranean constructed nests. Subsequently, exotrophic tadpoles finish their development in ponds. Similar to mode 30, species with reproductive mode 32 reproduce in subterranean constructed chambers. However, the endotrophic tadpoles complete their development in a nest.

In addition, the analysis showed that transitions from aquatic to terrestrial (or at least to less aquatic) reproductive modes have occurred at least four times in Leptodactylinae: 1) a shift from mode 11 to modes 30 or 32 in the ancestral *Adenomera* (nodes 1 and 2, Figures 2 and 3); 2) a shift from mode 30 to 32 in some *Adenomera* species (nodes 6 and 7); 3) a shift from mode 11 to 30 in some *Leptodactylus* ancestral (nodes 11 and 12); and a shift from 11 to 13 in two species of the *L. melanonotus* group (nodes 26 and 27). Moreover, transitions from terrestrial to aquatic (or at least a less terrestrial) reproductive modes were also found: 1) a shift from mode 32 to 30 in some *Adenomera* species (nodes 3 and 4); and 2) a shift from mode 30 to 13 in species of the *Leptodactylus pentadactylus* group (nodes 13 and 20). Besides those, transformation direction in node 2 was uncertain because it had equal probability for reproductive modes 30 and 32 (node 2, see Figure 3). Thus, the reproductive mode may have changed from 30 to 32 in node 3 or from 32 to 30 in node 10 (Figure 3).

While clutch size, tadpole environment, nuptial spines and egg pigmentations presented a clear evolutionary pattern with few independent origins of states (Figure 3), multiple reversals between ‘open areas’ and ‘forest formations’ were found by the habitat reconstruction. The most recent common ancestor of *Adenomera* and *Lithodytes* (node 1) produced less than 50 eggs per clutch and lacked nuptial spines and melanin on eggs, while the *Leptodactylus* most recent common ancestor (node 11) had large clutch sizes, tadpoles in lentic water bodies, presence of nuptial spines and the absence of egg pigmentation.

The character mapping analysis retrieved the estimated number of changes in the ancestral nodes, together with the probable transformations along the branches (Table 2). The habitat had the highest expected number of changes (approximately 32), being almost the same number from one state to another. The reproductive mode had 20 changes, most of them between modes 32 to 30. However, nuptial spine presented the lowest number of transformations (eight changes).

**Table 2:** **Evolutionary data for six Leptodactylinae life-history traits based on stochastic Bayesian character mapping**

**Character**	**Replications**	**Expected number of transformations**	**Expected number of character state transformation**												**Amount of time**				**Rate**
			**0–1**	**0–2**	**0–3**	**1–0**	**1–2**	**1–3**	**2–0**	**2–1**	**2–3**	**3–0**	**3–1**	**3–2**	**State 0**	**State 1**	**State 2**	**State 3**	
Reproductive mode	60,000	19.87	2.53	2.6	1.1	1.04	1.3	0.5	1.9	1.4	1.8	1	0.6	4.1	0.30	0.16	0.32	0.22	8.16
Clutch size	60,000	14.30	1.78	1.9	–	1.72	2.6	–	2.3	4	–	–	–	–	0.33	0.31	0.36	–	5.62
Habitat	60,000	31.60	16.4	–	–	15.2	–	–	–	–	–	–	–	–	0.46	0.54	–	–	11.8
Tadpole environment	60,000	16.39	1.83	1	–	2.66	4.8	–	1.5	4.6	–	–	–	–	0.08	0.69	0.23	–	6.33
Nuptial spines	60,000	7.94	2.91	–	–	5.03	–	–	–	–	–	–	–	–	0.58	0.42	–	–	2.64
Egg pigmentation	60,000	13.10	6.25	–	–	6.85	–	–	–	–	–	–	–	–	0.69	0.31	–	–	4.37

Estimated number of state transformations, amount of time and rate of transformation for each character for the 35 Leptodactylinae species based on stochastic Bayesian character mapping using 600 trees (See characters codes in Table 4).

The D statistics found significant correlations only between reproductive mode and clutch size or nuptial spines (Table 3). However, significant correlations between specific reproductive modes and other character states were also found (*dij* statistics; Table 3). For example, although reproductive mode and egg pigmentation presented no significant correlation (D = 0.47, p = 0.01), egg pigmentation had a positive association with reproductive modes 11 and 13 (respectively *dij* = 0.09, p = 1.0e-7 and 0.02, p = 1.0e-7), and a negative association with mode 32 (*dij* = −0.05, p =1.0e-7).

**Table 3:** **Correlation values obtained by the D and** ***dij*** **statistic for 35 Leptodactylinae species.**

	**Reproductive mode**		**Reproductive mode** ***dij***			
	***D***		**Mode 11**	**Mode 13**	**Mode 30**	**Mode32**
Clutch size	**0.72**	Less than 50	**−0.07**	**−0.04**	−0.01	**0.12**
		Between 50 and 1,000	**−**0.03	**−**0.01	**0.08**	**−0.05**
		More than 1,000	**0.09**	0.04	**−0.07**	**−0.07**
Habitat	0.30	Open areas	**−**0.01	**−**0.02	0.06	**−**0.03
		Forest formations	0.01	0.02	**−**0.06	0.03
Tadpole environment	0.49	Lotic water bodies	**−0.02**	**−0.01**	0.02	**–**
		Lentic water bodies	0.07	**0.03**	0.01	**–**
		Terrestrial	**–**	**–**	**–**	**–**
Nuptial spines	**0.65**	Absent	**−0.10**	**−**0.06	0.08	**0.08**
		Present	**0.10**	0.06	**−**0.08	**−0.08**
Egg pigmentation	0.47	Absent	**−0.09**	**−0.02**	0.06	**0.05**
		Present	**0.09**	**0.02**	−0.06	**−0.05**

Bolded values indicate p ≤ 0.05.

## Discussion

### Ancestral state reconstruction

Our results showed that the evolution of reproductive modes in Leptodactylinae did not follow a linear trend, as Heyer [15] predicted, and it did not necessarily happen through intermediate stages, as McDiarmid [14] suggested. Other interesting facts demonstrated here include the monophyletic independent sister lineages of *Adenomera* and *Leptodactylus* [17,18,20,23,31,32] and the independent origins of less aquatic modes, occurring in the ancestors of both genus, as suggested by Heyer [15].

Moreover, we found no indication of a gradual evolution of reproductive traits towards terrestriality through the lineages. Transitions were found from the most aquatic mode (11) to the semi-aquatic mode (30) and to the terrestrial mode (32) – both consists in eggs in foam nests deposited in burrows, but while in the first the tadpoles are carried by water to the water body, in the second the tadpoles develops entirely into the burrow. This same situation was demonstrated for other terrestrial modes of reproduction in anurans [13]. This shift from mode 11 to 30 may have happened two times: 1) from the *Leptodactylus* genus ancestor to the ancestor of the *L. fuscus* and *L. pentadactylus* groups; and 2) from the *Lithodytes* and *Adenomera* ancestor to the most recent common ancestor of the latter genus. In this last case, even with the confident phylogenetic position of *Lithodytes lineatus* (which was also shown by [17,18,21,31,32]), there is still uncertainty about its ancestor state (Figure 2). Its breeding site is uncommon in the Leptodactylinae species, with the parent laying eggs in foam nests inside ant nests. Besides, it is not clear whether or not the tadpoles subsequently complete their development in temporary water bodies. This reproductive mode does not fit any of those previously assigned to the subfamily.

The absence of a linear evolution from aquatic to terrestrial reproductive modes is confirmed by reversions from mode 30 – eggs in foam nests inside burrows and tadpoles subsequently carried to the water body - to mode 13 – eggs in foam nests in constructed basins near the water – and also from mode 32 – eggs in foam nests and complete development of the tadpoles inside burrows - to mode 30 (Figure 2). Reversions from terrestrial to aquatic reproductive modes have also been demonstrated in other anuran groups [13]. Also, the character mapping analysis showed that these transitions occurred not only at the most recent common hypothetic ancestor (at the nodes), but also along the branches. The transitions from a more terrestrial breeding mode to one that is less-so can also be observed by summing up the expected number of transformations in this direction and comparing them to the transformations from aquatic to terrestrial breeding (see Table 2). Both presented nearly 10 transformations, showing that terrestrial egg laying is not necessarily an evolutionary tendency, but is actually an alternative strategy with no implied directionality.

The analysis of ancestral state reconstruction of clutch size also showed evidence of a clear pattern among lineages. The ancestral state of the *Leptodactylus* species suggested the oviposition of more than 1,000 eggs per clutch, while *Adenomera* had less than 50 eggs (see Figure 3). Even though a reversion was found from in *Adenomera* from the reproductive mode which tadpoles develops inside the burrows (32) to the mode that tadpoles are carried from the burrow to the water body (30), the hypothetical ancestral state holds the oviposition of few eggs. It is unlikely that the exotrophic *Adenomera* tadpoles simply float to the water bodies by chance (e.g., due to topography or great rain incidence), because they have functional mouthparts and spiracles [33,34], implying that they need nourishment provided from the environment to complete their metamorphoses. Endotrophic tadpoles do not have mouthparts or spiracles, as they use only the yolk provided by their parents for their development.

### Character mapping and correlations

A clear association pattern between clutch size and reproductive mode was found, as predicted by Heyer [15] and previously demonstrated for many anuran genera [13]. The higher dependence on water, which is a more unpredictable environment when compared to a burrow, together with no parental care, favors r-strategists, which may allocate energy to increase the number of eggs per clutch. Conversely, species with a low dependency on water, in this case, egg-burrowing species, may allocate energy to parental care [35,36]. The correlation analyses showed that the ancestral state of *Leptodactylus* and *Adenomera* species presents both kinds of reproductive strategies, with the first favoring productivity, and the other favoring parental care.

The construction of the subterranean chambers by males of the *Leptodactylus fuscus* group and the *Adenomera* species is considered a type of parental care, with the parents providing a more suitable microhabitat for offspring development [14,37]. When compared to water environments, subterranean chambers increase the chance of offspring survivorship by reducing predation, desiccation and interspecific competition [14,38,39]. Egg-burrowing species have a limitation regarding the number of eggs due to both the amount of energy spent in the burrow construction, and the limited space inside the chamber [40]. Thus, space may be an important constraint, since terrestriality in anurans demands increased amounts of yolk to feed the endotrophic tadpoles, consequently increasing egg dimensions [15,40,41]. The amount of yolk needed in the reproductive mode which tadpoles complete the development inside the burrow (32) is higher than in mode in which they are carried to the water (30), because in the first case the tadpole completes the development exclusively using yolk as an energy source. Conversely, species with mode 30 only depend on the yolk for a brief period of tadpole development, which may lead to contrasting correlations of modes 32 and 30 and clutch sizes. An opposite relationship was noticed in the more aquatic reproductive mode, with tadpoles using external sources of energy from the beginning of development, leading to a smaller egg dimension and larger clutch size, and consequently, to a correlation of egg size and oviposition.

We found no evidence of a clear association with the phylogenetic hypothesis of Leptodactylinae and evolution in their habitat usage (open and forest formations), especially since this trait presented the highest number of expected transformations and no significant correlations with reproductive modes. The correlations provide no evidence to support the hypothesis that the evolution of terrestrial breeding is linked to forest habitats due to the high humidity [42,43]. However, air humidity may not limit the development of Leptodactylinae because the foam nest may protect eggs from desiccation*.* Although we are aware that the lack of correlation and the high number of transformations may be a consequence of the small number of categories (open and forest), there is not enough data available to be more specific about the habitat that these species use.

Environmental filters seem to have been decisive for the evolution of tadpole environment in *Leptodactylus*, since all species shared the same state: lentic water bodies. This tadpole environment appears in Leptodactylinae during almost 70% of the lineage reconstructions (see Table 2). Species with aquatic reproduction that involves placing eggs in lentic environments have an adaptive advantage when compared to those that use lotic water bodies because lentic waters facilitate the amplexus, providing a sheltered environment for eggs and the tadpole’s first stage of development, in addition to keeping the nest integrity. Consequently, the reproductive modes more related to aquatic breeding (11 and 13) are negatively correlated with lotic water bodies.

Our results showed similar evolutionary histories for nuptial spines and egg pigmentation. The presence or absence of these structures occurred together in almost the same species and ancestral nodes, with two major exceptions: the ancestor of *Leptodactylus* and the *L. pentadactylus* group. Both lacked egg pigmentation but had nuptial spines, which help the male anchor to the female. The presence of nuptial spines in *Leptodactylus*’ ancestors was maintained in the ancestors of the *Leptodactylus latrans* and *L. melanonotus* groups, including *L. discodactylus*, but is now associated with the presence of melanin on eggs. Although *Leptodactylus discodactylus* has not been assigned to any phenetic groups yet, our results suggest that it belongs to the *L. melanonotus* group (see [18,21,31]). The ancestral state of the *Leptodactylus pentadactylus* group, which had nuptial spines but lacked melanin on eggs, corroborates Heyer’s [15] hypothesis. He stated that the presence of spines in the *Leptodactylus pentadactylus* group has evolved because of the large adult body size of this species rather than because of the water dependence.

The species in this group do not place eggs directly in a main body of water. Spines are used to facilitate the amplexus between large specimens where eggs are placed in water accumulated in basins constructed by males. The lack of egg pigmentation in the group’s ancestors, which is only needed in eggs exposed to ultraviolet light, also supports Heyer’s hypothesis. Nevertheless, we found a positive correlation between mode 13 (eggs in constructed basins near the water), which is common among the *Leptodactylus pentadactylus* group and some species of the *Leptodactylus melanonotus* group, and egg pigmentation. This could be associated with the ability of species in the *Leptodactylus pentadactylus* and *Leptodactylus melanonotus* groups to construct basins where eggs are spawned [26]. In many cases, the eggs are exposed to ultraviolet light, and the egg pigmentation helps protect against embryo damage. The correlation analysis also confirmed the association between the presence of spines and egg pigmentation with the more aquatic reproductive mode (mode 11) and a negative correlation with the more terrestrial breeding (mode 32).

## Conclusions

Our results showed no evidence of an evolutionary tendency toward terrestriality in Leptodactylinae. Indeed, we found reversals from terrestrial to aquatic tadpole development and no evidence of mandatory intermediate stages. In addition, we also found correlations between morphological and ecological traits driven by water dependence. Aquatic reproductive modes are associated with higher clutch sizes, lentic waters, and the presence of nuptial spines and egg pigmentation. No correlation was found between reproductive modes and habitat usage, where multiple reversals of ancestors and descendants living in open and forested areas were found. The robustness of the phylogenetic hypothesis, which confirmed *Adenomera* and *Leptodactylus* monophyly and *Lithodytes* as a sister taxon of *Adenomera*, enabled the study of reproductive trait evolution. Furthermore, the present study reinforces the usefulness and power of Bayesian stochastic character mapping to better understand the evolution of life history traits.

## Methods

### Taxon sampling

We sampled 35 Leptodactylinae species, 11 *Adenomera*, and 23 *Leptodactylus,* representing all recognized phenetic groups: the *Leptodactylus latrans* group, the *Leptodactylus melanonotus* group, the *Leptodactylus pentadactylus* group, and the *Leptodactylus fuscus* group*,* as well as the monotypic *Lithodytes lineatus* (Additional file 3). *Physalaemus cuvieri* and *Physalaemus nattereri* were used as outgroups based on their relationships with Leptodactylinae [17,18]. Ninety-one sequences were obtained in this work and 40 were obtained from GenBank (Additional file 3).

Although our phylogenetic sampling does not represent the majority of leptodactyline species, we are confident that the results of character reconstruction, character mapping and correlation analyses will hold even if the remaining species were included in the phylogenetic hypothesis because: (i) most species with phylogenetic uncertainty (*Adenomera*) were included, (ii) these species were also the ones with higher variations in character states regarding reproductive modes; (iii) previous studies pointed out that *Leptodactylus* phenetic groups are phylogenetically structured, especially the *L. fuscus*, *L. melanonotus* (with *L. discodactylus*), and *L. latrans* [18,23,31,44], (iv) although the phylogenetic positioning of *L. pentadactylus* group was not yet certain, our results are in consonance with previous ones [18,31,32,44-47], and (v) most of the reproductive and morphological attributes of *Leptodactylus* were conserved among the phenetic groups [44,48]. In addition, natural history traits are not available for many leptodactyline species, which constrains the analysis of trait evolution.

### Genetic data

Total DNA was extracted from muscle or liver tissue preserved in ethanol and a tissue-storage buffer using the DNeasy Tissue Kit (Qiagen®). We sequenced four DNA fragments. The nuclear Rhodopsin exon I (*Rhod*) fragment was sequenced using Rhod1A and Rhod1C primers [49]. The mitochondrial regions 12S and 16S were sequenced using 12Sa, 12Sb, 16Sar and 16Sd [50]. For cytochrome B (c*ytB*), we used MVZ15 [51] and H15149 primers [52] (PCR protocols on Additional file 4). The PCR products were purified using 1.0 U of each enzyme, “shrimp alkaline phosphatase” (SAP) and exonuclease I (EXOI) (Biotech Pharmacon, ASA). Purified PCR products were sequenced in both directions on an ABI 3100 automated DNA sequencer (Applied Biosystems, CA) using the DYEnamic™ ET terminator sequencing kit (GE HealthCare, Sweden), according to the manufacturer’s instructions.

### Sequence alignment and phylogenetic analyses

The sequences were edited using SeqScape (v2.1) software and were then aligned in MUSCLE 3.8 [53]. The sequences that were not available were coded as missing data (Additional file 3). Coding sequences were tested for saturation plotting transitions and transversions against TN93 distance [54] using the DAMBE [55] software.

Phylogenetic hypotheses were obtained for the combined datasets using Bayesian and maximum-parsimony methods. Evolutionary model selection was performed using Akaike Information Criterion (AIC) implemented in jModelTest 2 [56]. Then, Bayesian analyses were conducted in MrBayes v.3.1.2 [57] with randomly generated starting trees. Four Markov Chains and four million generations were sufficient to obtain a standard deviation of split frequencies below 0.01. Trees and parameter values were sampled every 500 generations. After discarding the first 2500 trees (“burn-in”) of the two runs, we generated the 50% majority-rule consensus and calculated the Bayesian credibility values (BC) for each branch. Clades with BC equal to or exceeding 95% were considered strongly supported [58]. The maximum parsimony (MP) analysis was carried out using PAUP* 4.0 [59]. We used a heuristic search with multiple tree bisection reconnection (TBR) branch swapping. Bootstrap re-sampling [60] was applied to assess the support for individual clades using 1,000 bootstrap replicates and full heuristic searches with 10 replicates of random stepwise addition and TBR branch swapping. Clades with bootstrap values higher than 75% were considered well-supported following what indicated by [61].

### Ancestral state reconstruction, character mapping, and correlation analysis

To study the evolution of life-history traits among Leptodactylinae lineages, we inferred ancestral states, mapped character state changes and tested the correlation of six ecological and morphological traits using SIMMAP 1.5 [62]. At least four reproductive modes are known for Leptodactylinae [9]: (i) mode ‘11’ includes species that produce floating foam nests in ponds with exotrophic tadpoles; (ii) mode ‘13’ also presents exotrophic tadpoles, but with foam nests placed in water accumulated in constructed basins; (iii) mode ‘30’ groups species with foam nests placed inside a subterranean chamber, and after a period of development, the tadpoles float to bodies of water; and (iv) mode ‘32’ is the most terrestrial one, with endotrophic tadpoles (develop entirely in subterranean chambers using only the yolk as a source of energy) (Figure 1). Other life-history traits studied here were considered directly (clutch size, tadpole environment, nuptial spines and egg pigmentation) or indirectly (habitat) related to reproductive modes in frogs. Character states were retrieved from the literature (Additional file 2), based on personal observation or from specialists in the reproductive traits of Neotropical anurans (information by authority), and were coded as shown in Table 4.

**Table 4:** **Character codification used in the life-history trait analysis of Leptodactylinae**

**Character**	**State 0**	**State 1**	**State 2**	**State 3**
Reproductive mode	Mode 11	Mode 13	Mode 30	Mode 32
Clutch size	Less than 50	Between 50 and 1,000	More than 1,000	–
Habitat	Open areas	Forest formations	–	–
Tadpole environment	Lotic water bodies	Lentic water bodies	Terrestrial tadpole	–
Nuptial spine	Absence	Presence	–	–
Egg pigmentation	Absence	Presence	–	–

The character codification was used in the ancestral state reconstruction, character mapping and correlation analysis of the six life-history traits for 35 species of Leptodactylinae. Polymorphic data were coded as missing data.

We reconstructed the ancestral states of the six characters using Bayesian stochastic character mapping [8] on the 50% majority-rule consensus tree obtained from the Bayesian phylogenetic analysis (Additional file 5). The analysis evaluated the consistency between character history and character states observed at the tips, before then estimating the posterior probabilities of ancestral states. The results were visualized as pie charts using a function in the R software, developed by Dr. Marion Chartier. We also mapped the changes of character states along the phylogeny to estimate the number of transformations between states [6]. To perform this analysis, we randomly selected 600 trees from the Bayesian phylogenetic analysis after the convergence.

Afterwards we calculated the overall character correlation (D statistic) between reproductive mode and five life-history traits (i.e., clutch size, habitat, tadpole environment, nuptial spine, egg pigmentation), and the correlation state-by-state (*dij*). For this analysis, we randomly selected 300 trees generated after convergence by the Bayesian phylogenetic analysis. The *dij* statistic represents the divergence between the observed and expected association of states *i* and *j.* The expected association is the product of the marginal probabilities of finding these states (*i* and *j*) in the same phylogenetic node [7].

## Ethics statement

The Brazilian Institute for Biodiversity Conservation (Chico Mendes Institute for Biodiversity Conservation - ICMBio) provided a license under the number 021010.002153/05-11 to collect some of the specimens used in this study. Many sample tissues were also obtained by donation of other institutions (see Additional file 3). We confirm that all species used in this work are not endangered or protected species.

## Availability of supporting data

The data sets supporting the results of this article are available in the GenBank repository, http://www.ncbi.nlm.nih.gov/genbank, and can be accessed through the identifier indicated in the Additional file 3. The following data sets supporting the results of this article are available in the Dryad Digital repository, [http://dx.doi.org/10.5061/dryad.88kj7]: 1. Table containing the GenBank accession numbers of the sequences from *Adenomera*, *Leptodactylus* and *Lithodytes* used in phylogenetic. 2. Table with the character states for the six life-history traits (reproductive mode, clutch size, habitat, tadpole environment, nuptial pads or spines and egg pigmentation) for 35 Leptodactylinae species. 3. State probability of the six reconstructed life-history traits at each ancestral node of the 50% majority-rule consensus cladogram. 4. Life-history traits reconstructed were reproductive mode, clutch size, habitat, tadpole environment, nuptial pads or spines and egg pigmentation. 5. Nexus file used in MrBayes software to generate Bayesian molecular phylogenetic hypothesis [63].

## Additional files

Additional file 1:
**Saturation plot of the third codon positions for the Cytochrome B fragment of 35 Leptodactylinae species.** Transitions (indicated by triangles) and transversions (indicated by X) are plotted against the Tamura-Nei (1993) distance. Additional file 2:
**Character states used in evolutionary analyses and respective citation sources.**
Additional file 3:
**Leptodactylinae species sampled and GenBank accession numbers.**
Additional file 4:
**PCR protocols for the amplified fragments.**
Additional file 5:
**State probability of the reconstructed life-history traits at each ancestral node of the 50% majority-rule consensus cladogram.**
