# Supplementary figures and images for: Ancestral reconstruction of reproductive traits shows no tendency toward terrestriality in leptodactyline frogs
Source: BMC Evol Biol. 2015 May 20;15:91. doi: 10.1186/s12862-015-0365-6 (PMC4437749; doi:10.1186/s12862-015-0365-6)

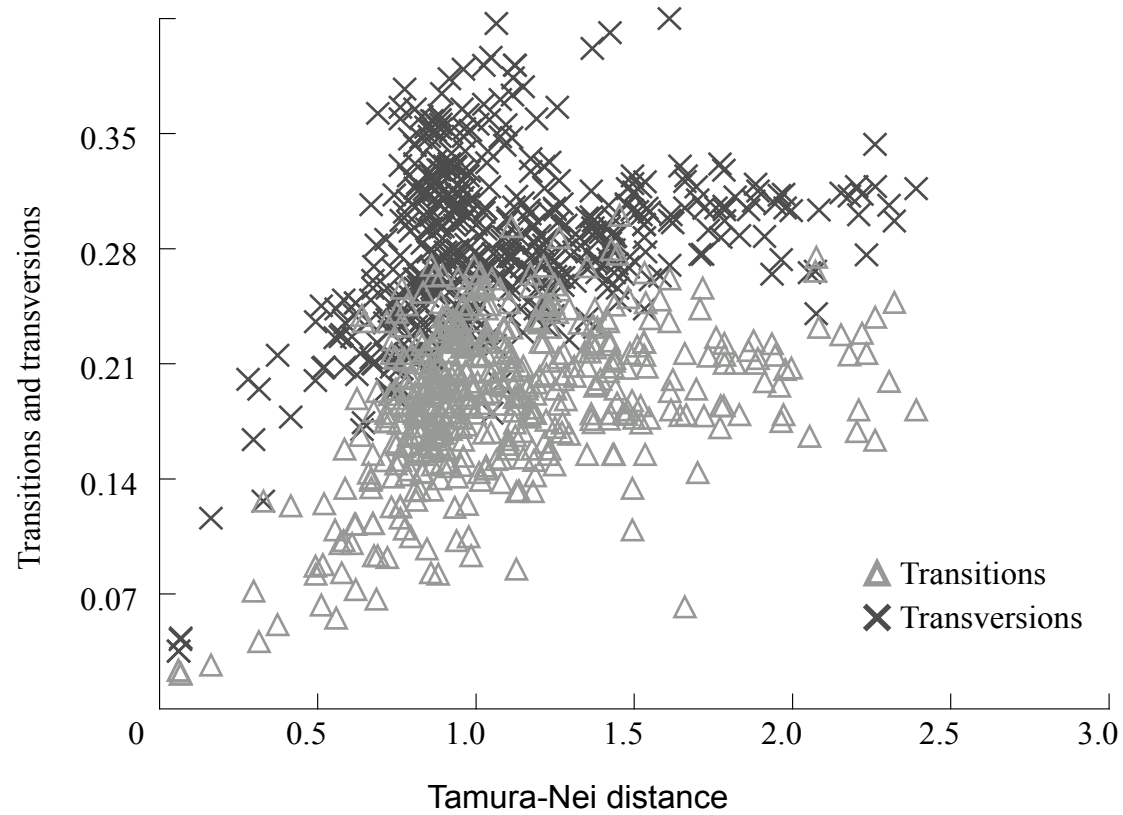

Supplement: Additional file 1: — Saturation plot of the third codon positions for the Cytochrome B fragment of 35 Leptodactylinae species. Transitions (indicated by triangles) and transversions (indicated by X) are plotted against the Tamura-Nei (1993) distance. [file 12862_2015_365_MOESM1_ESM.pdf]
