# Supplementary material for: Ancestral reconstruction of reproductive traits shows no tendency toward terrestriality in leptodactyline frogs
Source: BMC Evol Biol. 2015 May 20;15:91. doi: 10.1186/s12862-015-0365-6 (PMC4437749; doi:10.1186/s12862-015-0365-6)
Supplement: Additional file 2: — Character states used in evolutionary analyses and respective citation sources. [file 12862_2015_365_MOESM2_ESM.docx]

Additional file 2. Leptodactylinae species sampled and GenBank accession numbers.

| Species | Locality | Acronym and number | GenBank accession numbers | | | |
| --- | --- | --- | --- | --- | --- | --- |
|  | (Country: State: Municipality) |  | 16S | 12S | Cytochrome B | Rhodopsin 1 |
| *Adenomera andreae* | Brazil: Amazonas: Manaus | AAG-UFU 4283 | KC477261 | KC470112 | KF548069 | KF613599 |
| *Adenomera araucaria* | Brazil: Santa Catarina: Lebón Régis | MCP 9673 | KC477241 | KC470091 | KF548070 | KF613578 |
| *Adenomera bokermanni* | Brazil: Tocantins: Caseara | CHUNB 46021 | KC477243 | KC470107 | KF548072 | KF613580 |
| *Adenomera diptyx* | Brazil: Mato Grosso do Sul: Corumbá | LHUFCG 0173 | KC477249 | KC470099 | KF548078 | KF613587 |
| *Adenomera engelsi* | **-** | GenBank | **KC603940** | **KC603940** | **KC603970** | **KC604100** |
| *Adenomera heyeri* | **-** | GenBank | **KC603948** | **KC603947** | **KC603972** | **KC604096** |
| *Adenomera hylaedactyla* | Brazil: Amazonas: Manaus | AAG-UFU 4736 | KC477260 | KC470111 | KF548068 | KF613598 |
| *Adenomera lutzi* | Guyana | IRSNB 13951 | KC477251 | KC470100 | KF548079 | KF613588 |
| *Adenomera marmorata* | Brazil: São Paulo: Caraguatatuba | LHUFCG 0165 | KC477242 | KC470092 | KF548071 | KF613579 |
| *Adenomera saci* | Brazil: Distrito Federal: Brasília | AAG-UFU 4238 | KC477244 | KC470090 | KF548065 | KF613577 |
| *Adenomera thomei* | **-** | GenBank | **KC603946** | **KC603945** | **KC603971** | **KC604101** |
| *Physalaemus nattereri* | **-** | GenBank | **AY326020** | **AY326020** | **-** | **-** |
| *Leptodactylus albilabris* | **-** | GenBank | **EF091413** | **EF091410** | **EF091393** | **-** |
| *Leptodactylus chaquensis* | **-** | GenBank | **EF632055** | **AY943221** | **-** | **-** |
| *Leptodactylus discodactylus* | **-** | GenBank | **DQ283433** | **AY943226** | **-** | **DQ284033** |
| *Leptodactylus elenae* | Brazil: Mato Grosso do Sul: Corumbá | AAG-UFU 4211 | KC477248 | KC470098 | KF548077 | KF613586 |
| *Leptodactylus fallax* | **-** | GenBank | **EF091415** | **EF091412** | **EF091407** | **-** |
| *Leptodactylus furnarius* | Brazil: Minas Gerais: Paracatu | CHUNB 25860 | **-** | KC470108 | KF548085 | KF613595 |
| *Leptodactylus fuscus* | Brazil: Minas Gerais: Buritizeiro | LHUFCG 0491 | KC477246 | KC470095 | KF548074 | KF613583 |
| *Leptodactylus jolyi* | Brazil: Distrito Federal: Brasília | AAG-UFU 3124 | KC477250 | KC470093 | KF548066 | KF613581 |
| *Leptodactylus knudseni* | **-** | GenBank | **EF632056** | **EF613180** | **EF091409** | **-** |
| *Leptodactylus latrans* | **-** | GenBank | **-** | **AY143353** | **AY843934** | **AY844681** |
| *Leptodactylus labyrinthicus* | - | GenBank | **AY947860** | **AY947874** | **-** | **-** |
| *Leptodactylus leptodactyloides* | Brazil: Acre: Rio Branco | AAG-UFU 4199 | KC477247 | KC470096 | KF548075 | KF613584 |
| *Leptodactylus macrosternum* | Brazil: Tocantins: Vale do Paranã | AAG-UFU 2679 | KC477255 | KC470106 | KF548084 | KF613594 |
| *Leptodactylus marambaiae* | Brazil: Rio de Janeiro: Ilha de Marambaia | AAG-UFU 4193 | **-** | KC470097 | KF548076 | KF613585 |
| *Leptodactylus melanonotus* | **-** | GenBank | **DQ347060** | **AY943224** | **-** | **AY364405** |
| *Leptodactylus mystaceus* | Brazil: Acre: Rio Branco | AAG-UFU 4197 | KC477252 | KC470101 | KF548080 | KF613589 |
| *Leptodactylus mystacinus* | Brazil: Distrito Federal: Brasília | **-** | KC477256 | KC470105 | KF548067 | KF613593 |
| *Leptodactylus notoaktites* | Brazil: Santa Catarina: Itapema | AAG-UFU 3129 | KC477254 | KC470104 | KF548083 | KF613592 |
| *Leptodactylus petersii* | Brazil: Tocantins: Caseara | CHUNB45794 | **-** | KC470109 | KF548086 | KF613596 |
| *Leptodactylus podicipinus* | Brazil: Mato Grosso do Sul: Corumbá | LHUFCG 0244 | KC477245 | KC470094 | KF548073 | KF613582 |
| *Leptodactylus pustulatus* | Brazil: Tocantins: Palmas | CHUNB11258 | **-** | KC470110 | KF548087 | KF613597 |
| *Leptodactylus rhodomystax* | Brazil: Acre: Rio Branco | AAG-UFU 4196 | **AY947855** | KC470103 | KF548082 | KF613591 |
| *Leptodactylus rhodonotus* | **-** | GenBank | **EU368908** | **AM039727** | **EU368908** | **-** |
| *Lithodytes lineatus* | Brazil: Acre: Rio Branco | AAG-UFU 4198 | KC477253 | KC470102 | KF548081 | KF613590 |
| *Physalaemus cuvieri* | **-** | GenBank | JQ627212 | AY819347 | AY843975 | AY844717 |

Museum abbreviations: Coleção Herpetológica da Universidade de Brasília (CHUNB), Coleção Ariovaldo A. Giaretta da Universidade Federal de Uberlândia (AAG-UFU), Institut Royal des Sciences Naturelles de Belgique (IRSNB), Laboratório de Herpetologia da Universidade Federal de Campina Grande (LHUFCG), and Museu de Ciências e Tecnologia da Pontifícia Universidade Católica de Porto Alegre (MCP).
