# Supplementary material for: Ancestral reconstruction of reproductive traits shows no tendency toward terrestriality in leptodactyline frogs
Source: BMC Evol Biol. 2015 May 20;15:91. doi: 10.1186/s12862-015-0365-6 (PMC4437749; doi:10.1186/s12862-015-0365-6)
Supplement: Additional file 3: — Leptodactylinae species sampled and GenBank accession numbers. [file 12862_2015_365_MOESM3_ESM.docx]

Additional file 3. PCR protocols for the amplified fragments.

1. PCR mix components and quantities for each of the amplified fragments

| Reagent | 16S | 12S and *cytB* | *Rhod* |
| --- | --- | --- | --- |
| Deionized water | 7.7 µL | 8.5 µL | 7.5 µL |
| DNA | 2.0 µL | 2.0 µL | 3.0 µL |
| Forward primer (2 mM) | 3.0 µL | 3.0 µL | 3.0 µL |
| Reverse primer (2mM) | 3.0 µL | 3.0 µL | 3.0 µL |
| Buffer 1X* | 2.0 µL | 2.0 µL | 2.0 µL |
| DNTPs (2,5 mM) | 2.0 µL | 1.2 µL | 1.2 µL |
| Taq polymerase (5u/µL) | 0.3 µL | 0.3 µL | 0.3 µL |
| Total volume | 20 µL | 20 µL | 20 µL |

*Buffer 1X (10 mM Tris-HCl, pH 8.3, 50 mM KCl, 1.5 mM MgCl2)

1. PCR thermal program for each of the amplified fragments

| Fragment | Initial heating | Denaturation | Annealing | Extension | Final extension |
| --- | --- | --- | --- | --- | --- |
|  |  | 34 cycles | | |  |
| 16S | 2 min at 94ºC | 60s at 94ºC | 60s at 54ºC - 58ºC | 90s at 72ºC | 10 min at 72ºC |
|  |  | 35 cycles | | |  |
| 12S, *cytB* and *Rhod* | 2 min at 94ºC | 60s at 94ºC | 60s at 54ºC - 58ºC | 60s at 72ºC | 6 min at 72ºC |
