# Supplementary material for: Ancestral reconstruction of reproductive traits shows no tendency toward terrestriality in leptodactyline frogs
Source: BMC Evol Biol. 2015 May 20;15:91. doi: 10.1186/s12862-015-0365-6 (PMC4437749; doi:10.1186/s12862-015-0365-6)
Supplement: Additional file 4: — PCR protocols for the amplified fragments. [file 12862_2015_365_MOESM4_ESM.docx]

Table S3. Character states for the six life-history traits for 35 Leptodactylinae species. Characters coded as in Table 1.

| **Species** | **Reproductive mode** | | **Cluch size** | | **Habitat** | | **Tadpole environment** | | **Nuptial pads or spines** | | **Egg pigmentation** | |
| --- | --- | --- | --- | --- | --- | --- | --- | --- | --- | --- | --- | --- |
|  | **State** | **Reference** | **State** | **Reference** | **State** | **Reference** | **State** | **Reference** | **State** | **Reference** | **State** | **Reference** |
| *Adenomera andreae* | 3 | [1] | 0 | [1] | 1 | [1] | 2 | [2] | 0 | [3] | 0 | [1] |
| *Adenomera araucaria* | 3 | [4] | ? | - | 1 | [4] | 2 | [4] | 0 | [4] | 0 | Personal observation |
| *Adenomera bokermanni* | 2 | [5] | 0 | [6] | 0 | [6] | ? | - | 0 | [3] | 0 | [3] |
| *Adenomera diptyx* | 2 | [7] | 0/1 | [7] | 0 | [8] | 0 | [8] | ? | - | 0 | Personal observation |
| *Adenomera engelsi* | 3 | [9] | ? | - | 1 | [10] | ? | - | 0 | [10] | ? | - |
| *Adenomera heyeri* | ? | - | ? | - | 1 | [11] | ? | - | 0 |  | 0 | Personal observation |
| *Adenomera hylaedactyla* | 3 | [1] | 0 | [1] | 0 | [12] | 2 | [13] | 0 | [3] | 0 | [1] |
| *Adenomera lutzi* | ? | - | 0 | [14] | 1 | [14] | ? | - | 0 | [14] | 0 | Personal observation |
| *Adenomera marmorata* | 3 | [3] | 0 | [15] | 0/1 | [15] | 2 | [15] | 0 | [16] | 0 | [16] |
| *Adenomera saci* | 2 | Personal observation | 0 | [3] | 0 | Personal observation | 0 | Personal observation | 0 | [3] | 0 | [3] |
| *Adenomera thomei* | 2 | [17] | 0 | [17] | 1 | [17] | ? | - | 0 | [17] | 0 | [17] |
| *Leptodactylus albilabris* | 2 | [18] | ? | - | ? | - | ? | - | 0 | [19] | 0 | [20] |
| *Leptodactylus chaquensis* | 0 | [5] | 2 | [3] | 0 | [8] | 1 | [16] | 1 | [16] | 1 | [16] |
| *Leptodactylus discodactylus* | ? | - | 1/2 | [21] | 1 | [1] | 1 | [16] | 0 | [22] | 0 | [21] |
| *Leptodactylus elenae* | 2 | [23] | ? | - | 0 | [8] | 1 | [23] | 0 | [16] | ? | - |
| *Leptodactylus fallax* | ? | - | 2 | [24] | 1 | [24] | 2 | [25] | 1 | [16] | ? | - |
| *Leptodactylus furnarius* | 2 | [26] | 0/1 | [27] | 0 | [27] | 1 | [28] | 0 | [29] | 0 | [16] |
| *Leptodactylus fuscus* | 2 | [5] | 1 | [30] | 0 | [31] | 1 | [31] | 0 | [16] | 0 | [16] |
| *Leptodactylus jolyi* | 2 | [26] | ? | - | ? | - | 0/1 | [26] | 0 | [16] | ? | - |
| *Leptodactylus knudseni* | 1 | [1] | 1 | [32] | 1 | [1] | 1 | [33] | 1 | [1] | 0 | Lima, AP |
| *Leptodactylus latrans* | 0 | [5] | 2 | [9] | 0 | [8] | 1 | [18] | 1 | [15] | 1 | [9] |
| *Leptodactylus labyrinthicus* | 1 | [34] | 2 | [35] | 0 | [36] | 1 | [37] | 1 | [37] | 1 | [35] |
| *Leptodactylus leptodactyloides* | 0 | [18] | 1/2 | [38] | 0/1 | [8] | 1 | [16] | 1 | [3] | 1 | [16] |
| *Leptodactylus macrosternum* | 0 | [5] | 2 | [39] | 0 | [8] | 1 | [18] | 1 | [16] | 1 | Lima, AP |
| *Leptodactylus marambaiae* | ? | - | ? | - | ? | - | ? | - | 0 | [16] | ? | - |
| *Leptodactylus melanonotus* | 0 | [3] | 2 | [3] | ? | - | 1 | [16] | 1 | [16] | 1 | [16] |
| *Leptodactylus mystaceus* | 2 | [3] | 1 | [1] | 0/1 | [36] | 1 | Menin, M | 0 | [1] | 0 | [3] |
| *Leptodactylus mystacinus* | 2 | [18] | 1 | [40] | 0 | [31] | 1 | [31] | 0 | [16] | 0 | [40] |
| *Leptodactylus notoaktites* | 2 | [5] | ? | - | ? | - | 0 | [33] | 0 | [16] | ? | - |
| *Leptodactylus petersii* | 1 | [32] | 2 | Rodrigues, DJ | 1 | [8] | 1 | [18] | 1 | [16] | ? | - |
| *Leptodactylus podicipinus* | 1 | [18] | 2 | [18] | 0 | [8] | 1 | [18] | 1 | [16] | 1 | [18] |
| *Leptodactylus pustulatus* | 0 | [18] | ? | - | 1 | [41] | 1 | [41] | 1 | [16] | 1 | [42] |
| *Leptodactylus rhodomystax* | 1 | [1] | 1 | [1] | 1 | [8] | 1 | [37] | 1 | [1] | 0 | Lima, AP |
| *Leptodactylus rhodonotus* | ? | - | ? | - | 1 | [8] | 1 | [37] | 1 | [1] | ? | - |
| *Lithodytes lineatus* | ? | - | 1 | [43] | 1 | [8] | 1 | [16] | 0 | [3] | 0 | [1] |

1. Rodriguez LO, Duellman WE. Quitos region, Amazonian Peru; 1994.

2. Hero J. An Illustrated key to tadpoles occurring in the Central Amazon rainforest, Manaus, Amazona, Brazil. Amazoniana 1990;11:201 – 262.

3. Heyer WR. Relationship of the *marmoratus* species group (Amphibia, Leptodactylidae) within the subfamily leptodactylinae. Contrib Sci. 1974;253:1–45.

4. Kwet A, Angulo A. A new species of *Adenomera* (Anura, Leptodactylidae) from Araucaria forest of Rio Grande do Sul (Brazil), with comments on the systematic status of southern populations of the genus. Alytes. 2002;20:28–43.

5. Haddad C, Prado C. Reproductive modes in frogs and their unexpected diversity in the Atlantic Forest of Brazil. Bioscience. 2005;55:207–217.

6. Heyer WR. Systematics of the *marmoratus* group of the frog genus *Leptodactylus* (Amphibia, Leptodactylidae). Contrib Sci. 1973.

7. De La Riva I. A new reproductive mode for the genus *Adenomera* (Amphibia: Anura: Leptodactylidae): taxonomic implications for certain Bolivian and Paraguayan populations. Stud Neotrop Fauna Environ. 1995;30:15–29.

8. De La Riva I, Köhler J, Lötters S, Reichle S. Ten years of research on Bolivian amphibians: updated checklist, distribution, taxonomic problems, literature and iconography. Rev Española Herpetol. 2000;14:19–164.

9. Kwet A, Di-Bernardo M. Pró-Mata. Anﬁbios – Amphibien – Amphibians. 1st edition. Porto Alegre: EDIPUCRS; 1999.

10. Kwet A, Steiner J, Zillikens A. A new species of *Adenomera* (Amphibia: Anura: Leptodactylidae) from the Atlantic rain forest in Santa Catarina, southern Brazil. Stud Neotrop Fauna Environ. 2009;44:93–107.

11. Boistel R, Massary J De, Angulo A. Description of A new species of the genus *Adenomera* (Amphibia, Anura, Leptodactylidae) from French Guiana. Acta Herpetol. 2006;1–14.

12. Menin M, Almeida AP De, Kokubum MNDC. Reproductive aspects of *Leptodactytus* *hylaedactylus* (Anura: Leptodactylidae), a member of the Leptodactylus marmoratus species group , with a description of tadpoles and calls. J Nat Hist. 2009;43:2257–2270.

13. Heyer WR, Silverstone PA. The larva of the frog *Leptodactylus* *hylaedactylus* (Leptodactylidae). Fieldiana Zool. 1969;51:141 – 145.

14. Kok PJR, Kokubum MNC, Macculloch RD, Lathrop A. Morphological variation in *Leptodactylus* *lutzi* (Anura, Leptodactylidae) with description of its advertisement call and notes on its courtship behavior. Phyllomedusa. 2007;6:45–60.

15. Heyer WR, Rand AS, Cruz CAG, Peixoto OL, Nelson CE. Frogs of Boracéia. Arq Zool. 1990;31:231–410.

16. Heyer WR. The relationships of *Leptodactylus diedrus* (Anura, Leptodactylidae). Alytes. 1998;16:1–24.

17. Almeida A de P, Angulo A. A new species of *Leptodactylus* (Anura: Leptodactylidae) from the state of Espírito Santo, Brazil, with remarks on the systematics of associated populations. Zootaxa. 2006;1334:1–25.

18. Prado C de A, Uetanabaro M, Haddad CFB. Description of a new reproductive mode in *Leptodactylus* (Anura, Leptodactylidae), with a review of the reproductive specialization toward terrestriality in the genus. Copeia. 2002;2002:1128–1133.

19. Maxson LR, Heyer WR. Molecular systematics of the frog genus *Leptodactylus* (Amphibia: Leptodactylidae). Zoology. 1988;1:1–8.

20. Dent JN. Observations of the life history and development of *Leptodactylus albilabris*. Copeia. 1956;4:207–210.

21. Heyer WR, Bellin MS. Ecological notes on five sympatric *Leptodactylus* (Amphibia, Leptodactylidae) from Ecuador. Herpetologica1. 1973;29:66–72.

22. Heyer WR. Studies on frogs of the genus *Leptodactylus* (Amphibia, Leptodactylidae). VI. Biosystematics of the m*elanonotus* group. Los Angeles Cty Museum Nat Hist. 1970;1:1 – 50.

23. Prado CPA, D’Heursel A. The tadpole of *Leptodactylus* *elenae* (Anura : Leptodactylidae), with the description of the internal buccal anatomy the tadpole of *Leptodactylus* *elenae* (Anura : Leptodactylidae ), with the description of the internal. South Am J Herpetol. 2006;1:79–86.

24. Daltry JC. Mountain chicken monitoring manual; 2002.

25. Gibson RC, Buley KR. Maternal care and obligatory oophagy in *Leptodactylus fallax*: a new reproductive mode in frogs. Copeia. 2004;1:128–135.

26. Sazima I, Bokermann WCA. Cinco novas espécies de *Leptodactylus* do centro e sudeste brasileiro (Amphibia, Anura, Leptodactylidae). Rev Bras Biol. 1978;38:899–912.

27. Giaretta AA, Kokubum MNDC. Reproductive ecology of *Leptodactylus furnarius* Sazima & Bokermann, 1978, a frog that lays eggs in underground chambers (Anura: Leptodactylidae). Herpetozoa. 2004;16:115–126.

28. McDiarmid RW, Altig R: Tadpoles. The Biology of Anuran Larvar. Volume 2000. Chicago, Illinois: University of Chicago Press; 1999.

29. Heyer WR, Heyer MH. *Leptodactylus furnarius* Sazima and Bokermann. 2004;1:1–5.

30. Lucas E, Brasileiro C, Oyamaguchi H, Martins M. The reproductive ecology of *Leptodactylus fuscus* (Anura, Leptodactylidae): new data from natural temporary ponds in the Brazilian Cerrado and a review throughout its distribution. J Nat Hist. 2008;42:2305–2320.

31. De-Carvalho CB, Freitas EB, Faria RG, Batista R de C, Batista C de C, Coelho WA, Bocchiglieri A. História natural de *Leptodactylus mystacinus* e *Leptodactylus fuscus* (Anura: Leptodactylidae) no Cerrado do Brasil Central. Biota Neotrop. 2008;8:105 – 116.

32. Lima AP, Magnusson WE, Menin M, Erdtmann L, Rodrigues DDJ, Keller C, Hödl W. Guia de sapos da reserva Adolpho Ducke, Amazônia Central. 1st edition. Manaus, Brazil: Attema Design Editorial; 2006.

33. Sá RO De, Langone JA, Segalla M V. The tadpole of *Leptodactylus notoaktites* Heyer, 1978. 2007;1978:69–75.

34. Agostinho CA. Caracterização de populações de rã-pimenta *Leptodactylus labyrinthicus* (Spix, 1824) e avaliação de seu desempenho em cativeiro. Universidade Federal de São Carlos, São Paulo, Brazil; 1994.

35. Zina J, Haddad CFB. Reproductive activity and vocalizations of *Leptodactylus labyrinthicus* (Anura: Leptodactylidae) in southeastern Brasil. Biota Neotrop. 2005;5:1–11.

36. Giaretta AA, Menin M, Facure KG, Kokubum MNDC, Filho JCDO. Species richness, relative abundance, and habitat of reproduction of terrestrial frogs in the Triângulo Mineiro region, Cerrado biome, southeastern Brazil. Iheringia Série Zool. 2008;98:181–188.

37. Eterovick P, Sazima I. Description of the tadpole of *Leptodactylus syphax*, with a comparison of morphological and ecological characters of tadpoles and adults of the species in the *L*. *pentadactylus* group (Leptodactylidae, Anura). Amphibia-Reptilia. 2000;21:341–350.

38. Duellman WE. Cusco Amazónico: The lives of amphibians and reptiles in an Amazonian Rainforest; 2005.

39. Uetanabaro M, Prado CPA, Rodrigues DDJ, Gordo M, Campos Z. Guia de campo dos anuros do pantanal sul e planaltos de entorno. 1st edition. UFMS and UFMT; 2008.

40. Oliveira Filho JC De, Giaretta AA. Reproductive behavior of *Leptodactylus mystacinus* (Anura, Leptodactylidae) with notes on courtship call of other *Leptodactylus* species. Iheringia Série Zool. 2008;98:508–515.

41. De Sá RO, Brandão RA, Guimarães LD. Description of the tadpole of *Leptodactylus pustulatus* Peters, 1870 (Anura: Leptodactylidae). Zootaxa. 2007;58:49 – 58.

42. Fenolio DB, Silva HLR, Junior NJS. *Leptodactylus* *pustulatus* Peters, 1870 (Amphibia: Leptodactylidae): notes on habitat, ecology, and color in life. Herpetol Rev. 2006;37:140–142.

43. Bernarde PS, Kokubum MNDC. Seasonality, age structure and reproduction of *Leptodactylus* (*Lithodytes*) *lineatus* (Anura, Leptodactylidae) in Rondônia state, southwestern Amazon, Brazil. Iheringia Série Zool. 2009;99:368–372.
