# Supplementary material for: Ancestral reconstruction of reproductive traits shows no tendency toward terrestriality in leptodactyline frogs
Source: BMC Evol Biol. 2015 May 20;15:91. doi: 10.1186/s12862-015-0365-6 (PMC4437749; doi:10.1186/s12862-015-0365-6)
Supplement: Additional file 5: — State probability of the reconstructed life-history traits at each ancestral node of the 50% majority-rule consensus cladogram. [file 12862_2015_365_MOESM5_ESM.docx]

Additional file 5. Ancestral character state probabilities of each clade of the Bayesian cladogram of the 35 Leptodactilinae species. Bolded values indicate the highest probabilities.

| Character | Clade | Pr(0) | Pr(1) | Pr(2) | Pr(3) |
| --- | --- | --- | --- | --- | --- |
| Reproductive mode | 1 | **0.582** | 0.011 | 0.297 | 0.110 |
| Reproductive mode | 2 | 0.026 | 0.006 | 0.478 | **0.490** |
| Reproductive mode | 3 | 0.000 | 0.000 | 0.123 | **0.876** |
| Reproductive mode | 4 | 0.000 | 0.000 | **0.650** | 0.349 |
| Reproductive mode | 5 | 0.000 | 0.000 | **0.993** | 0.007 |
| Reproductive mode | 6 | 0.001 | 0.000 | **0.718** | 0.281 |
| Reproductive mode | 7 | 0.000 | 0.000 | 0.018 | **0.982** |
| Reproductive mode | 8 | 0.000 | 0.000 | 0.000 | **1.000** |
| Reproductive mode | 9 | 0.001 | 0.000 | 0.142 | **0.856** |
| Reproductive mode | 10 | 0.041 | 0.015 | **0.649** | 0.296 |
| Reproductive mode | 11 | **0.675** | 0.022 | 0.289 | 0.015 |
| Reproductive mode | 12 | 0.068 | 0.055 | **0.859** | 0.018 |
| Reproductive mode | 13 | 0.001 | 0.019 | **0.975** | 0.006 |
| Reproductive mode | 14 | 0.000 | 0.000 | **1.000** | 0.000 |
| Reproductive mode | 15 | 0.000 | 0.000 | **1.000** | 0.000 |
| Reproductive mode | 16 | 0.000 | 0.000 | **1.000** | 0.000 |
| Reproductive mode | 17 | 0.000 | 0.000 | **1.000** | 0.000 |
| Reproductive mode | 18 | 0.000 | 0.000 | **1.000** | 0.000 |
| Reproductive mode | 19 | 0.000 | 0.000 | **1.000** | 0.000 |
| Reproductive mode | 20 | 0.005 | **0.672** | 0.142 | 0.181 |
| Reproductive mode | 21 | 0.000 | **0.998** | 0.001 | 0.001 |
| Reproductive mode | 22 | **0.998** | 0.001 | 0.001 | 0.000 |
| Reproductive mode | 23 | **1.000** | 0.000 | 0.000 | 0.000 |
| Reproductive mode | 24 | **1.000** | 0.000 | 0.000 | 0.000 |
| Reproductive mode | 25 | **0.998** | 0.002 | 0.001 | 0.000 |
| Reproductive mode | 26 | **0.661** | 0.302 | 0.036 | 0.002 |
| Reproductive mode | 27 | 0.000 | **0.999** | 0.000 | 0.000 |
| Reproductive mode | 28 | **0.900** | 0.078 | 0.021 | 0.002 |
| Reproductive mode | 29 | **0.508** | 0.051 | 0.347 | 0.094 |
| Clutch size | 1 | **0.742** | 0.170 | 0.088 | **-** |
| Clutch size | 2 | **0.999** | 0.001 | 0.000 | **-** |
| Clutch size | 3 | **1.000** | 0.000 | 0.000 | **-** |
| Clutch size | 4 | **1.000** | 0.000 | 0.000 | **-** |
| Clutch size | 5 | **1.000** | 0.000 | 0.000 | **-** |
| Clutch size | 6 | **1.000** | 0.000 | 0.000 | **-** |
| Clutch size | 7 | **1.000** | 0.000 | 0.000 | **-** |
| Clutch size | 8 | **0.994** | 0.002 | 0.004 | **-** |
| Clutch size | 9 | **0.994** | 0.002 | 0.004 | **-** |
| Clutch size | 10 | **0.997** | 0.001 | 0.002 | **-** |
| Clutch size | 11 | 0.007 | 0.063 | **0.931** | **-** |
| Clutch size | 12 | 0.010 | 0.389 | **0.601** | **-** |
| Clutch size | 13 | 0.002 | **0.960** | 0.038 | **-** |
| Clutch size | 14 | 0.001 | **0.997** | 0.002 | **-** |
| Clutch size | 15 | 0.002 | **0.994** | 0.003 | **-** |
| Clutch size | 16 | 0.012 | **0.970** | 0.018 | **-** |
| Clutch size | 17 | 0.000 | **1.000** | 0.000 | **-** |
| Clutch size | 18 | 0.017 | **0.957** | 0.026 | **-** |
| Clutch size | 19 | 0.320 | 0.202 | **0.478** | **-** |
| Clutch size | 20 | 0.007 | 0.271 | **0.722** | **-** |
| Clutch size | 21 | 0.006 | 0.372 | **0.623** | **-** |
| Clutch size | 22 | 0.000 | 0.001 | **0.999** | **-** |
| Clutch size | 23 | 0.000 | 0.000 | **1.000** | **-** |
| Clutch size | 24 | 0.000 | 0.000 | **1.000** | **-** |
| Clutch size | 25 | 0.000 | 0.001 | **0.999** | **-** |
| Clutch size | 26 | 0.002 | 0.002 | **0.997** | **-** |
| Clutch size | 27 | 0.000 | 0.000 | **1.000** | **-** |
| Clutch size | 28 | 0.040 | 0.241 | **0.719** | **-** |
| Clutch size | 29 | 0.306 | 0.280 | **0.414** | **-** |
| Habitat | 1 | 0.091 | **0.909** | **-** | **-** |
| Habitat | 2 | 0.021 | **0.979** | **-** | **-** |
| Habitat | 3 | 0.058 | **0.942** | **-** | **-** |
| Habitat | 4 | **0.633** | 0.367 | **-** | **-** |
| Habitat | 5 | **0.998** | 0.002 | **-** | **-** |
| Habitat | 6 | 0.047 | **0.953** | **-** | **-** |
| Habitat | 7 | 0.014 | **0.986** | **-** | **-** |
| Habitat | 8 | 0.024 | **0.976** | **-** | **-** |
| Habitat | 9 | 0.221 | **0.779** | **-** | **-** |
| Habitat | 10 | 0.007 | **0.993** | **-** | **-** |
| Habitat | 11 | 0.435 | **0.565** | **-** | **-** |
| Habitat | 12 | 0.287 | **0.713** | **-** | **-** |
| Habitat | 13 | **0.569** | 0.431 | **-** | **-** |
| Habitat | 14 | **0.997** | 0.003 | **-** | **-** |
| Habitat | 15 | **0.970** | 0.030 | **-** | **-** |
| Habitat | 16 | 0.211 | **0.789** | **-** | **-** |
| Habitat | 17 | **0.998** | 0.002 | **-** | **-** |
| Habitat | 18 | **0.997** | 0.003 | **-** | **-** |
| Habitat | 19 | **0.996** | 0.004 | **-** | **-** |
| Habitat | 20 | 0.067 | **0.933** | **-** | **-** |
| Habitat | 21 | 0.056 | **0.944** | **-** | **-** |
| Habitat | 22 | **0.738** | 0.262 | **-** | **-** |
| Habitat | 23 | **0.993** | 0.007 | **-** | **-** |
| Habitat | 24 | **1.000** | 0.000 | **-** | **-** |
| Habitat | 25 | 0.141 | **0.859** | **-** | **-** |
| Habitat | 26 | 0.024 | **0.976** | **-** | **-** |
| Habitat | 27 | 0.363 | **0.637** | **-** | **-** |
| Habitat | 28 | 0.073 | **0.927** | **-** | **-** |
| Habitat | 29 | 0.290 | **0.710** | **-** | **-** |
| Tadpole environment | 1 | 0.002 | **0.962** | 0.036 | **-** |
| Tadpole environment | 2 | 0.019 | 0.489 | **0.492** | **-** |
| Tadpole environment | 3 | 0.015 | 0.025 | **0.960** | **-** |
| Tadpole environment | 4 | 0.076 | 0.027 | **0.897** | **-** |
| Tadpole environment | 5 | **0.853** | 0.091 | 0.056 | **-** |
| Tadpole environment | 6 | 0.011 | 0.146 | **0.843** | **-** |
| Tadpole environment | 7 | 0.000 | 0.044 | **0.956** | **-** |
| Tadpole environment | 8 | 0.000 | 0.005 | **0.995** | **-** |
| Tadpole environment | 9 | 0.003 | **0.681** | 0.316 | **-** |
| Tadpole environment | 10 | 0.015 | **0.782** | 0.204 | **-** |
| Tadpole environment | 11 | 0.000 | **1.000** | 0.000 | **-** |
| Tadpole environment | 12 | 0.000 | **1.000** | 0.000 | **-** |
| Tadpole environment | 13 | 0.000 | **0.998** | 0.002 | **-** |
| Tadpole environment | 14 | 0.000 | **1.000** | 0.000 | **-** |
| Tadpole environment | 15 | 0.000 | **1.000** | 0.000 | **-** |
| Tadpole environment | 16 | 0.017 | **0.982** | 0.001 | **-** |
| Tadpole environment | 17 | 0.000 | **1.000** | 0.000 | **-** |
| Tadpole environment | 18 | 0.000 | **1.000** | 0.000 | **-** |
| Tadpole environment | 19 | 0.000 | **1.000** | 0.000 | **-** |
| Tadpole environment | 20 | 0.000 | **0.977** | 0.022 | **-** |
| Tadpole environment | 21 | 0.000 | **0.999** | 0.001 | **-** |
| Tadpole environment | 22 | 0.000 | **1.000** | 0.000 | **-** |
| Tadpole environment | 23 | 0.000 | **1.000** | 0.000 | **-** |
| Tadpole environment | 24 | 0.000 | **1.000** | 0.000 | **-** |
| Tadpole environment | 25 | 0.000 | **1.000** | 0.000 | **-** |
| Tadpole environment | 26 | 0.000 | **1.000** | 0.000 | **-** |
| Tadpole environment | 27 | 0.000 | **1.000** | 0.000 | **-** |
| Tadpole environment | 28 | 0.000 | **1.000** | 0.000 | **-** |
| Tadpole environment | 29 | 0.007 | **0.956** | 0.037 | **-** |
| Nuptial spines | 1 | **0.964** | 0.036 | **-** | **-** |
| Nuptial spines | 2 | **1.000** | 0.000 | **-** | **-** |
| Nuptial spines | 3 | **1.000** | 0.000 | **-** | **-** |
| Nuptial spines | 4 | **1.000** | 0.000 | **-** | **-** |
| Nuptial spines | 5 | **0.999** | 0.001 | **-** | **-** |
| Nuptial spines | 6 | **1.000** | 0.000 | **-** | **-** |
| Nuptial spines | 7 | **1.000** | 0.000 | **-** | **-** |
| Nuptial spines | 8 | **0.999** | 0.001 | **-** | **-** |
| Nuptial spines | 9 | **0.999** | 0.001 | **-** | **-** |
| Nuptial spines | 10 | **0.999** | 0.001 | **-** | **-** |
| Nuptial spines | 11 | 0.012 | **0.988** | **-** | **-** |
| Nuptial spines | 12 | 0.015 | **0.985** | **-** | **-** |
| Nuptial spines | 13 | 0.395 | **0.605** | **-** | **-** |
| Nuptial spines | 14 | **1.000** | 0.000 | **-** | **-** |
| Nuptial spines | 15 | **1.000** | 0.000 | **-** | **-** |
| Nuptial spines | 16 | **0.999** | 0.001 | **-** | **-** |
| Nuptial spines | 17 | **0.999** | 0.001 | **-** | **-** |
| Nuptial spines | 18 | **1.000** | 0.000 | **-** | **-** |
| Nuptial spines | 19 | **1.000** | 0.000 | **-** | **-** |
| Nuptial spines | 20 | 0.011 | **0.989** | **-** | **-** |
| Nuptial spines | 21 | 0.001 | **0.999** | **-** | **-** |
| Nuptial spines | 22 | 0.000 | **1.000** | **-** | **-** |
| Nuptial spines | 23 | 0.001 | **0.999** | **-** | **-** |
| Nuptial spines | 24 | 0.000 | **1.000** | **-** | **-** |
| Nuptial spines | 25 | 0.000 | **1.000** | **-** | **-** |
| Nuptial spines | 26 | 0.005 | **0.995** | **-** | **-** |
| Nuptial spines | 27 | 0.001 | **0.999** | **-** | **-** |
| Nuptial spines | 28 | 0.004 | **0.996** | **-** | **-** |
| Nuptial spines | 29 | **0.557** | 0.443 | **-** | **-** |
| Egg pigmentation | 1 | **0.986** | 0.014 | **-** | **-** |
| Egg pigmentation | 2 | **1.000** | 0.000 | **-** | **-** |
| Egg pigmentation | 3 | **1.000** | 0.000 | **-** | **-** |
| Egg pigmentation | 4 | **1.000** | 0.000 | **-** | **-** |
| Egg pigmentation | 5 | **1.000** | 0.000 | **-** | **-** |
| Egg pigmentation | 6 | **1.000** | 0.000 | **-** | **-** |
| Egg pigmentation | 7 | **1.000** | 0.000 | **-** | **-** |
| Egg pigmentation | 8 | **0.999** | 0.001 | **-** | **-** |
| Egg pigmentation | 9 | **0.992** | 0.008 | **-** | **-** |
| Egg pigmentation | 10 | **0.999** | 0.001 | **-** | **-** |
| Egg pigmentation | 11 | **0.598** | 0.402 | **-** | **-** |
| Egg pigmentation | 12 | **0.865** | 0.135 | **-** | **-** |
| Egg pigmentation | 13 | **0.995** | 0.005 | **-** | **-** |
| Egg pigmentation | 14 | **1.000** | 0.000 | **-** | **-** |
| Egg pigmentation | 15 | **0.996** | 0.004 | **-** | **-** |
| Egg pigmentation | 16 | **0.967** | 0.033 | **-** | **-** |
| Egg pigmentation | 17 | **0.999** | 0.001 | **-** | **-** |
| Egg pigmentation | 18 | **1.000** | 0.000 | **-** | **-** |
| Egg pigmentation | 19 | **0.998** | 0.002 | **-** | **-** |
| Egg pigmentation | 20 | **0.917** | 0.083 | **-** | **-** |
| Egg pigmentation | 21 | **0.840** | 0.160 | **-** | **-** |
| Egg pigmentation | 22 | 0.006 | **0.994** | **-** | **-** |
| Egg pigmentation | 23 | 0.001 | **0.999** | **-** | **-** |
| Egg pigmentation | 24 | 0.000 | **1.000** | **-** | **-** |
| Egg pigmentation | 25 | 0.002 | **0.998** | **-** | **-** |
| Egg pigmentation | 26 | 0.016 | **0.984** | **-** | **-** |
| Egg pigmentation | 27 | 0.014 | **0.986** | **-** | **-** |
| Egg pigmentation | 28 | 0.088 | **0.912** | **-** | **-** |
| Egg pigmentation | 29 | **0.881** | 0.119 | **-** | **-** |
